# Supplementary material for: Optical Rotation—A Reliable Parameter for Authentication of Honey?
Source: Molecules. 2022 Dec 15;27(24):8916. doi: 10.3390/molecules27248916 (PMC9785960; doi:10.3390/molecules27248916)
Supplement: Supplementary file 1 [file molecules-27-08916-s001.zip › molecules-2067391-supplementary.pdf]

Table S1. Correlation matrix presenting the Pearson's coefficients (r) for 42 examined components *vs.* optical rotation angle.

	[α]	G	F	Gb	IMa	IMu	Kb	Lu	Ma	Mu	Ng	Su	ααTr	αβTr	Tru	Tu	Er	1-Ks	Mr	Mz	Pa	Rf
**[α]**	1																					
**G**	*0.09**	1																				
**F**	-0.89**	*-0.17*	1																			
**Gb**	*-0.22*	*-0.20*	*0.28*	1																		
**IMa**	*-0.20*	*-0.45*	*0.23*	*0.09*	1																	
**IMu**	*-0.19*	*-0.37*	*0.22*	*0.01*	0.71	1																
**Kb**	*-0.54*	*-0.29*	*0.52*	*0.14*	0.67	*0.57*	1															
**Lu**	*-0.42*	*-0.33*	*0.37*	*-0.03*	0.76	0.60	0.79	1														
**Ma**	0.88	*0.03*	-0.69	*-0.19*	*-0.32*	*-0.29*	-0.60	*-0.49*	1													
**Mu**	*-0.44*	*-0.41*	*0.39*	*0.05*	0.87	0.70	0.77	0.86	*-0.51*	1												
**Ng**	*-0.50*	*-0.33*	*0.45*	*0.20*	0.82	*0.57*	0.81	0.80	*-0.53*	0.89	1											
**Su**	*0.15*	*-0.36*	*-0.39*	*0.03*	*-0.17*	*-0.12*	*-0.18*	*-0.21*	*-0.09*	*-0.19*	*-0.25*	1										
**ααTr**	*-0.16*	*0.04*	*0.07*	*0.05*	*0.18*	*0.35*	*0.32*	*0.50*	*-0.26*	*0.30*	*0.31*	*-0.11*	1									
**αβTr**	-0.58	*-0.45*	*0.50*	*0.09*	0.76	*0.52*	0.74	0.77	-0.57	0.86	0.90	*-0.14*	*0.27*	1								
**Tru**	*-0.38*	*-0.33*	*0.42*	*0.13*	0.88	0.67	0.84	0.81	*-0.51*	0.80	0.76	*-0.20*	*0.24*	0.68	1							
**Tu**	*-0.62*	*-0.42*	*0.56*	*0.10*	0.66	*0.48*	0.66	0.67	*-0.53*	0.82	0.80	*-0.22*	*0.16*	0.88	0.61	1						
**Er**	*-0.35*	*-0.30*	*0.35*	*0.17*	*0.20*	*0.01*	*0.11*	*0.19*	*-0.25*	*0.27*	*0.26*	*-0.11*	*0.11*	*0.35*	*0.15*	*0.50*	1					
**1-Ks**	*-0.21*	*-0.09*	*0.12*	*-0.01*	*0.51*	*0.31*	*0.45*	*0.67*	*-0.33*	*0.55*	*0.46*	*-0.14*	*0.52*	*0.44*	*0.48*	*0.25*	*0.00*	1				
**Mr**	0.89	*0.03*	-0.72	*-0.21*	*-0.33*	*-0.27*	-0.62	*-0.50*	0.97	*-0.54*	*-0.57*	*-0.04*	*-0.24*	-0.61	*-0.51*	-0.59	*-0.27*	*-0.30*	1			
**Mz**	*-0.07*	*-0.13*	*0.06*	*-0.02*	*0.11*	*0.23*	*0.07*	*0.21*	*-0.13*	*0.14*	*0.11*	*-0.06*	0.60	*0.10*	*0.10*	*0.10*	*0.43*	*0.37*	*-0.08*	1		
**Pa**	*-0.08*	*-0.30*	*0.05*	*0.01*	*0.43*	*0.40*	*0.21*	*0.43*	*-0.22*	*0.37*	*0.33*	*0.01*	*0.54*	*0.32*	*0.39*	*0.30*	*0.53*	*0.35*	*-0.16*	0.82	1	
**Rf**	*-0.48*	*-0.55*	*0.38*	*0.11*	0.67	*0.49*	0.59	0.67	*-0.55*	0.74	0.67	*0.12*	*0.22*	0.78	0.61	0.66	*0.28*	0.59	-0.58	*0.27*	*0.43*	1

* Data without statistical significance at level p>0.05 are given in italics

** High positive correlations (r>0.8) are highlighted in red, and strong negative correlations (r<-0.8) - in blue.

Table S2. Honey and jam samples investigated

Sample	Type	Botanical origin	Geographical origin	Specific rotation $[\alpha]_D^{20}$
**dl1**	nectar	diluted	Bulgaria	+7.2
**dl2**	honeydew	diluted	Bulgaria	+31.1
**dl3**	nectar	diluted	Romania	+89.2
**dl4**	nectar	diluted	Romania	+34.1
**dl5**	nectar	diluted	Romania	+34.1
**dl6**	nectar	diluted	Romania	+46.1
**dl7**	nectar	diluted	Bulgaria	+39.3
**ch8**	mixed	chestnut	Bulgaria	-11.5
**ch9**	mixed	chestnut	Bulgaria	-3.2
**ch10**	mixed	chestnut	Bulgaria	-2.5
**ch11**	mixed	chestnut	Bulgaria	+18.4
**cf12**	honeydew	coniferous	Bulgaria	-6.3
**cf13**	honeydew	coniferous	Italy	+9.0
**cf14**	honeydew	coniferous	Romania	-8.1
**ok15**	honeydew	oak	Bulgaria	-9.7
**ok16**	honeydew	oak	Bulgaria	-11.3
**ok17**	honeydew	oak	Bulgaria	-10.4
**ok18**	honeydew	oak	Bulgaria	-2.9
**ok19**	honeydew	oak	Bulgaria	-7.4
**ok20**	honeydew	oak	Bulgaria	-8.6
**ok21**	honeydew	oak	Bulgaria	-10.6
**ok22**	honeydew	oak	North Macedonia	-0.2
**ok23**	honeydew	oak	Romania	-9.4
**ok24**	honeydew	oak	Romania	-4.6
**ts25**	nectar	thistle	Romania	+2.5
**ts26**	nectar	thistle	Bulgaria	-18.8
**tm27**	nectar	thyme	Bulgaria	-8.3
**cr28**	nectar	coriander	Bulgaria	-9.4
**rs29**	nectar	rapeseed	Bulgaria	-11.2
**pf30**	nectar	polyfloral	Greece	-16.9

**pf31**	nectar	polyfloral	Bulgaria	-14.8
**ac32**	nectar	acacia	Bulgaria	-14.6
**ac33**	nectar	acacia	Bulgaria	-14.1
**ac34**	nectar	acacia	Bulgaria	-19.2
**ld35**	nectar	linden	Bulgaria	-16.3
**ld36**	nectar	linden	Romania	-15.8
**mx37**	mixed	mixed	Bulgaria	-14.4
**mx38**	mixed	mixed	Bulgaria	-9.3
**mx39**	mixed	mixed	North Macedonia	-15.0
**mx40**	mixed	mixed	Romania	-11.3
**mx41**	mixed	mixed	Romania	-35.2
**jm42**	jam	jam	Bulgaria	+22.7
